# Quinoa: A Promising Crop for Resolving the Bottleneck of Cultivation in Soils Affected by Multiple Environmental Abiotic Stresses

**DOI:** 10.3390/plants13152117

**Published:** 2024-07-31

**Authors:** Zahra Dehghanian, Mohammad Ahmadabadi, Behnam Asgari Lajayer, Vahideh Gougerdchi, Mohsen Hamedpour-Darabi, Nazila Bagheri, Ritika Sharma, Ramesh R. Vetukuri, Tess Astatkie, Bernard Dell

**Affiliations:** 1 Department of Biotechnology, Faculty of Agriculture, Azarbaijan Shahid Madani University, Tabriz 53714-161, Iran; zdehganian@yahoo.com (Z.D.); ahmadabadiir@yahoo.com (M.A.); bagheri12255@gmail.com (N.B.); 2 Faculty of Agriculture, Dalhousie University, Truro, NS B2N 5E3, Canada; astatkie@dal.ca; 3 Department of Plant Breeding and Biotechnology, Faculty of Agriculture, University of Tabriz, Tabriz 5166616471, Iran; vahan1987@yahoo.com; 4 Department of Horticultural Science, Faculty of Agriculture, Shiraz University, Shiraz 7194684471, Iran; mhamedpour@googlemail.com; 5 Department of Botany, Central University of Jammu, Rahya Suchani, Samba, Jammu 181143, India; ritikabot.rsh@gndu.ac.in; 6 Department of Plant Breeding, Swedish University of Agricultural Sciences, 23422 Lomma, Sweden; ramesh.vetukuri@slu.se; 7 Centre for Crop and Food Innovation, Murdoch University, Murdoch 6150, Australia; b.dell@murdoch.edu.au

**Keywords:** abiotic stresses, quinoa, gene expression, molecular approaches, physiological responses

## Abstract

Quinoa (*Chenopodium quinoa* Willd.) has gained worldwide recognition for its nutritional values, adaptability to diverse environments, and genetic diversity. This review explores the current understanding of quinoa tolerance to environmental stress, focusing on drought, salinity, heat, heavy metals, and UV-B radiation. Although drought and salinity have been extensively studied, other stress factors remain underexplored. The ever-increasing incidence of abiotic stress, exacerbated by unpredictable weather patterns and climate change, underscores the importance of understanding quinoa’s responses to these challenges. Global gene banks safeguard quinoa’s genetic diversity, supporting breeding efforts to develop stress-tolerant varieties. Recent advances in genomics and molecular tools offer promising opportunities to improve stress tolerance and increase the yield potential of quinoa. Transcriptomic studies have shed light on the responses of quinoa to drought and salinity, yet further studies are needed to elucidate its resilience to other abiotic stresses. Quinoa’s ability to thrive on poor soils and limited water resources makes it a sustainable option for land restoration and food security enterprises. In conclusion, quinoa is a versatile and robust crop with the potential to address food security challenges under environmental constraints.

## 1. Introduction

Abiotic stress factors are a major cause of agricultural productivity decline, resulting in more than a 50% yield reduction worldwide [1]. Many abiotic stresses frequently coexist in nature. Plants have been extensively studied in relation to the main abiotic stressors, including drought, waterlogging, salinity, heavy metals, intense heat, frost, and UV-B radiation [1,2]. The consequences of climate change are expected to cause global average air temperatures to rise at a rate of 0.3 to 0.7 °C every decade, with a maximum increase of 4.8 °C by the end of this century [3]. Extreme temperature occurrences, especially summer heat waves, have drawn more attention due to the potential for serious effects on human health, and economic and environmental stability [4]. Quinoa seeds are a highly nutritious food that is high in protein, essential amino acids, fiber, vitamins, and minerals. They have a crunchy texture, a nutty flavor, and a low glycemic index [5]. This plant is native to the Andean region of South America, where it has been cultivated for thousands of years. It is widely grown from sea level to 4000 m in altitude [6]. Quinoa is traditionally classified into five distinct ecotypes based on geographic adaptation: (I) The Valley Ecotype, grown at elevations between 2000 and 3500 m above sea level (m.a.s.l.) in Colombia, Ecuador, Peru, and Bolivia; (II) the Altiplano Ecotype, grown at high altitudes > 3500 m.a.s.l. around Lake Titicaca on the border of Bolivia and Peru; (III) the Salares Ecotype, grown in the salt flats of Bolivia and Chile, which has a high tolerance to salinity; (IV) the Sea-level Ecotype, grown in the low-altitude areas of southern and central Chile; and (V) the Subtropical or Yungas Ecotype, grown in Bolivia’s low-altitude, humid valleys, which includes late-flowering genotypes [7]. It is thought that quinoa was domesticated in southern Peru and Bolivia, close to Lake Titicaca. There are shreds of evidence of quinoa cultivation as early as 7500–8000 years ago [8]. 

**Figure 1:**
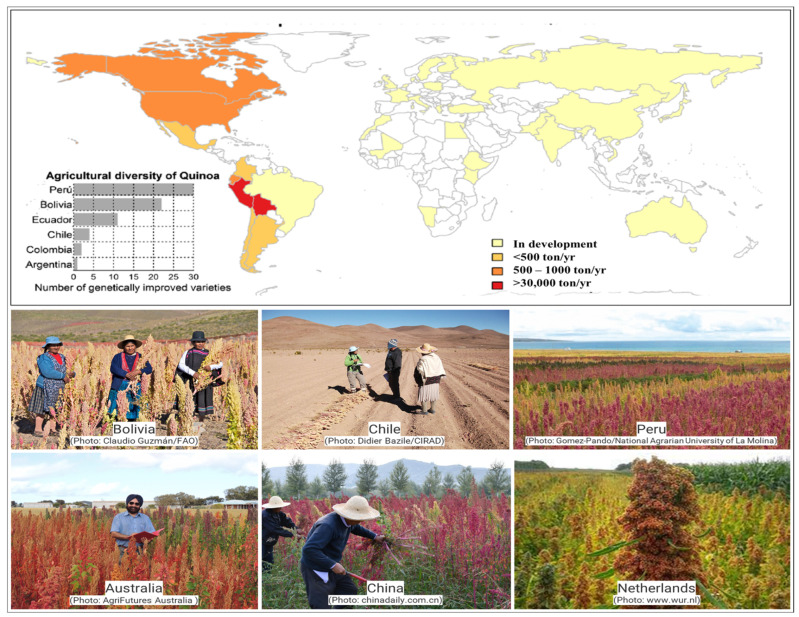
Quinoa production in various environmental conditions in different countries. The data on the number of genetically improved (elite cultivars bred for desirable traits) cultivars was reported in 2014. These cultivars are elite (modified from Ruiz et al. [20] and Ahmadzai [21]).

Because of the inherent variety in its germplasm, quinoa may adapt to a wide range of habitats. Traits include variations in inflorescence type, seed color, seed size, life-cycle duration, salinity tolerance, saponin content, and nutritional value [9,10]. Since the 1960s, several gene banks have been established in the Andes to preserve the genetic diversity of quinoa. Currently, there are 16,422 quinoa accessions conserved in 59 gene banks located in 30 countries, primarily in Bolivia and Peru [11]. The remarkable variety of quinoa with a wide range of characteristics allows it to flourish in a variety of conditions. This variation is essential for developing better quinoa cultivars that can endure challenging environments and produce larger yields [12]. Quinoa is a perfect crop for restoring degraded lands in hot, dry regions because of its resistance to drought, salinity, and poor soils. It is appropriate for areas with limited water supplies because of its deep root system which facilitates access to sub-soil moisture as well as efficient water usage [13]. Due to the significance of quinoa’s genetic diversity, gene banks have been established worldwide, which maintain an extensive collection of quinoa accessions to ensure the preservation of the genetic variety of this crop for future generations [14]. New approaches for enhancing quinoa’s resistance to abiotic stresses, including heat, salt, and drought stress, have been made possible by recent developments in genetics and molecular biology. These advancements hold promise for developing stress-resistant quinoa varieties that can thrive in a wider range of environments. Additionally, they can be used to create cultivars with higher stress tolerance and yield potential [15]. Figure 1 shows quinoa growing in various environmental conditions in different countries. Quinoa thrives in a wide range of poor soils, particularly those where soil sodicity and low soil moisture impede the growth of other grain crops. Several classical studies have investigated salinity and water deficit stress in quinoa [16]. Following the publication of quinoa’s genome sequence [17], transcriptomic investigations into drought and salinity in quinoa have been performed. However, little attention has been paid to quinoa’s tolerance to other abiotic stresses. Overall, quinoa stands out as a versatile and resilient crop with a promising future in sustainable agriculture [18]. Its adaptability to harsh environments, nutritional value, and genetic diversity make it a valuable resource for addressing food security challenges and restoring degraded lands. Further research and development are required to fully exploit the potential of quinoa and harness its benefits for humanity [19].

## 2. Examining the Adaptability of Quinoa under Stress Conditions 

Quinoa’s tolerance to multiple environmental circumstances has been attributed to the presence of numerous ecotypes that have evolved in diverse agro-environments [22]. Quinoa exhibits a variety of physiological and morphological adaptations that allow it to respond to drought and water deficits through avoidance, tolerance, and resistance mechanisms [23]. These adaptations include root and leaf development alterations, as well as ontogenetic variations [6]. Quinoa’s ability to endure abiotic stress is influenced by a variety of parameters at the tissue and plant levels. These elements include physiological, molecular, architectural, and morphological aspects [6]. Understanding these pathways is crucial for enhancing quinoa’s resistance to harsh environmental conditions. In addition to its adaptability, quinoa has considerable potential to address current and future concerns. It serves as a valuable source of genes for utilization in a variety of biotechnological applications. By harnessing the genetic diversity of quinoa, researchers can develop new crop varieties with enhanced stress tolerance and other desirable features. The following sections explore drought, salinity, and high temperature stress in detail as they are the areas where there has been most research activity over recent decades. This is followed by a smaller section that addresses other stresses such as low temperature and heavy metals.

## 3. Drought Stress

Before exploring drought in quinoa, it is useful to briefly consider drought responses in plants in general. Drought is defined in agricultural terms as an inadequate amount of moisture in the soil that affects the productivity of all crops [24,25]. The timing of soil drying in the life cycle of a crop can have significant consequences on crop yield and quality. In general, plants under drought stress undergo morphological and physiological changes, which significantly reduce crop yields. Agricultural drought considerably impacts the global food supply and is one of the most serious obstacles to sustainable agriculture [26]. In quinoa, the flowering and milky seed stages are the least drought-tolerant phases of plant development [27]. Studies on crop species in general have revealed that water deficit universally affects metabolic activities, including respiration, sugar metabolism, and photosynthesis [28]. It also diminishes cellular water potential, which affects cell elongation and growth. Plants that are stressed by drought release free radicals and reactive oxygen species (ROS), which raises the ethylene concentration [29]. Moreover, drought stress causes physiological alterations in crop plants and influences mineral nutrition by triggering a reduction in iron uptake in response to drought. Further reduction in iron uptake results in reduced zinc and manganese absorption, which is correlated with the altered expression of transport-associated genes [30]. Drought tolerance in plants refers to their capacity to withstand water deprivation and quickly recover growth post-rehydration. Plant growth and development are significantly impacted by drought, although during rehydration, certain activities including photosynthesis are restored [31]. 

The response of quinoa to drought reveals the presence of resilience traits. Manaa et al. [32] investigated the stress response of quinoa crops to water deprivation and discovered that quinoa plants exposed to dry conditions for one to two weeks experienced reduced growth, without any typical stress-induced impairments such as leaf curling, discoloration, or necrotic symptoms. In addition, their study revealed that rehydration after short drought periods largely restores any loss in plant yield. However, only plants that had been exposed to drought stress for one week recovered completely following two weeks of rehydration. Understanding the drought response mechanisms in quinoa may help to improve food production under climate change in the future.

### 3.1. Drought Response Mechanisms

**Figure 2:**
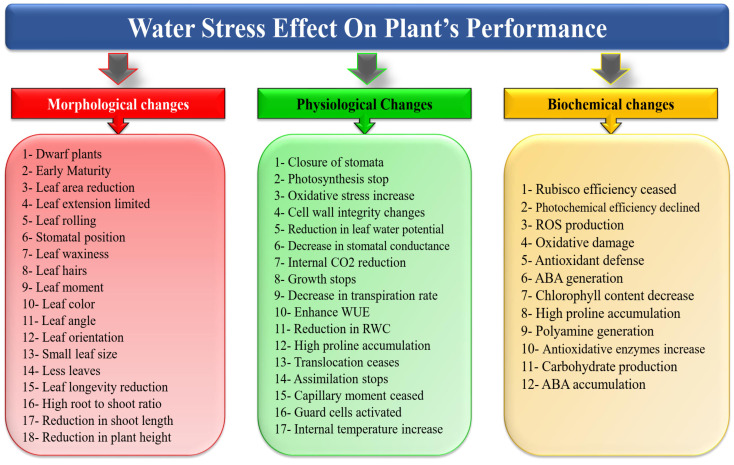
Impact of water stress on the morphological, physiological, and biochemical dynamics of plants [35].

The general strategies that plants employ to cope with drought-induced water constraints are summarized in Figure 2 and have been discussed in the literature [33,34,35,36]. It has been shown that quinoa genotypes differ in their physiological responses to drought stress in terms of CO_2_ assimilation rates, chlorophyll fluorescence, and seed yield [33]. Under drought stress, quinoa increases its water absorption capacity by promoting root development. In addition, it produces more proline and soluble sugars to preserve osmotic balance and adjust to water-deficit conditions. In quinoa leaves, drought stress causes a decrease in total chlorophyll content as well as an increase in H_2_O_2_ and malondialdehyde (MDA) content, which are signs of oxidative damage [34]. Understanding these pathways is crucial for developing crop varieties with enhanced drought tolerance and resilience.

The significant role of ABA in plant–water balance and development is well-established [36]. Under drought stress, quinoa plants (variety ‘INIA-Illpa’) had elevated levels of ABA in their roots, as reported by Kaur et al. [37]. In addition, leaves of the ‘Titicaca’ sea-level variety had high ABA levels when grown under both water-deficient and controlled conditions [38]. Similar results have been reported previously [39,40]. Furthermore, during drought stress, the concentration of ABA in the xylem of quinoa variety ‘Titicaca’ increased more rapidly in the shoots than in the roots [38]. The concentration of zylem ABA in ‘Titicaca’ and the altiplano variety ‘Achachino’ followed similar trajectories, rising two days after drought treatment and falling to control levels following re-watering. However, under drought conditions, ‘Titicaca’ had higher ABA concentrations than ‘Achachino’ [41]. 

Zurita et al. [6] discovered that the quinoa altiplano variety ‘Kankolla’ is consistently tolerant to drought, particularly in terms of the osmotic stress response during early vegetative growth. Their conclusion is based on the principle that elevated net photosynthetic rates and specific leaf area during early growth stages improve water absorption by larger root systems, empowering ‘Kankolla’ to withstand dry conditions. Quinoa, like many other crops, is more susceptible to water stress at particular growth phases. Under Bolivian Altiplano conditions, drought response mechanisms may include a delay in development when water deficiency is imposed during the pre-anthesis stage, including flowering, and grain development phases [42]. In quinoa, water stress during the pre-anthesis stage can accelerate flowering and shorten the seed-filling stage [33]. At this reproductive stage, drought can have a major effect on plant development and productivity [43]. 

A primary consequence of drought stress on plants is a decrease in the photosynthetic rate, which is largely attributed to reduced stomatal conductance [44]. Leaf gaseous exchange (∆) and carbon isotope discrimination are commonly utilized to investigate crop responses to drought stress [45]. Zurita et al. [6] measured ∆^13^C in 10 quinoa varieties cultivated all over the arid highlands of northwest Argentina, an area that typically receives a total of 160 mm of rainfall across the planting period [6]. Their findings showed that quinoa varieties with enhanced stomatal conductance sustained higher rates of photosynthesis. They also noticed a positive correlation between ∆^13^C and yield, revealing substantial diversity in grain production among cultivars. Furthermore, Killi and Haworth [46] discovered that drought-induced quinoa exhibits considerable stomatal and mesophyll CO_2_ transport limits. In greenhouse experiments with sea-level quinoa variety Red Head, indicators of leaf photosynthetic capacity, such as the maximum quantum yield of PSII (Fv/Fm) and quenching analysis (qP and qN), were generally not sensitive to water stress. However, a greenhouse study with ‘Titicaca’ revealed that Fv/Fm decreased in response to drought stress [38].

Over two consecutive growing seasons in Morocco, Ferroni et al. [47] used fast chlorophyll a fluorescence induction (OJIP) analysis to investigate changes in the photochemical performance of photosystem II (PSII) in quinoa under drought stress in field conditions. The OJIP transient, defined by the O, J, I, and P steps corresponding to the redox states of PSI and PSII, has been shown to yield comparable results to established methods [47] and was used to evaluate the impact of drought. The OJIP fluorescence transient analysis involves exposing a dark-adapted plant sample to high irradiance and measuring the resulting fluorescence transient. The fluorescence intensity is measured at specific time points: Fo (O-step), initial fluorescence at 50 μs; Fj (J-step), fluorescence at 2 ms; Fi (I-step), fluorescence at 30 ms; and Fm (P-step), maximum fluorescence intensity [47]. Results from a study on a sea-level grown ‘Puno’ quinoa variety revealed that the maximum quantum yield of PSII (Fv/Fm) and the quantum yield of electron transport (φE0) decreased due to drought stress [48]. These results suggest that OJIP parameters can serve as viable tools for assessing drought stress in quinoa. However, another investigation of chlorophyll fluorescence OJIP transient in the sea-level variety of quinoa ‘Red Head’, grown in Italy under semi-controlled conditions, revealed no significant differences between the control and drought treatments in 16 parameters associated with chlorophyll fluorescence [46]. These contradicting findings probably demonstrate that quinoa responds differently to drought depending on its genotype. Further research is needed to determine the effectiveness of chlorophyll fluorescence OJIP transient as a drought assessment tool for quinoa. A larger number of genotypes and simultaneous measurements of gas exchange should be included in these investigations. 

Recent studies have investigated the root system architecture of quinoa and its relatives, with a focus on soil moisture conditions. Compared to its close relatives, the wet habitat-adopted *C. hiricinum* and the dry environment-suited *C. pallidicaule*, quinoa (*C. quinoa*) roots exhibited faster elongation as well as more abundant and longer external branching, leading to improved foraging capacity [49]. Another study on root architecture and dynamics under drought stress showed that taproots develop more rapidly in the dry-habitat *C. quinoa* genotype compared to *C. hiricinum* and *C. pallidicaule*. In addition, the *C. quinoa* genotype had longer, coarser, and more branched root architecture [50]. Based on these results, the authors suggested using quinoa as a plant model to investigate the biophysical and ecophysiological properties of plant root systems in deeper soil layers. 

### 3.2. Drought Stress Management 

Apart from irrigation, various techniques have been suggested to mitigate the effects of drought stress on quinoa. Nitrogen (N) as ammonium nitrate (NH_4_NO_3_) (0.6 g N/ha) has been shown in greenhouse studies with “Titicaca” to improve quinoa performance under drought stress. Some key drought tolerance mechanisms observed in quinoa include the following: faster stomatal closure, lower leaf water potential, and higher leaf ABA concentrations [51]. According to a recent study on quinoa, the addition of organic waste and acidified biochar to drying soils could benefit crop physiology and yield, as well as improve the biochemical and chemical characteristics of the seed [52]. When 10 t ha^−1^ compost was applied in non-irrigated areas, quinoa yields in Morocco increased from 1.7 to 2.0 t ha^−1^, demonstrating how organic additions might mitigate the effects of drought stress [53]. In Chile’s semi-arid environment, the addition of vermicompost increased the yield of two quinoa genotypes [54]. In a pot trial under drought stress and optimal water supply conditions, applying acidified biochar to the soil improved quinoa seed production by 62% [55]. Furthermore, a study conducted on the quinoa genotype ‘V9’ under a range of irrigation conditions revealed that foliar treatments of 150 mg L^−1^ synthetic ascorbic acid and a 25% concentration of orange juice (a natural source of ascorbic acid) diluted in distilled water mitigated the negative effects of drought stress on quinoa [52]. Applying exogenous ascorbic acid has also been shown to benefit some other crop species [55].

Furthermore, foliar applications of 12.5 and 25.0 mM proline improved growth parameters, relative water content, yield components, and nutritional values of quinoa under field conditions in Egypt [55,56]. For instance, the application of 25.0 mM proline increased the yield from 6.23 g plot^−1^ to 8.56 g plot^−1^ under drought conditions. Moreover, a greenhouse pot experiment conducted using the sea-level quinoa cultivar ‘Pichaman’ demonstrated that quinoa growth is improved in arid environments by priming seeds with 80 mM exogenous H_2_O_2_ as well as foliar spraying with 15 mM H_2_O_2_ [57]. Chlorophyll, proline, sugar, and ABA concentrations rose along with photosynthesis and stomatal conductivity as a result of these treatments [57]. In *Vigna radiata*, external H_2_O_2_ has been shown to serve as an oxidative modulator in facilitating the release of stored proteins [58]. 

Quinoa’s unique fungal–root interactions may also enhance its drought tolerance [59]. A genomic study revealed that a wide variety of endophytic fungi, such as *Penicillium*, *Phoma*, and *Fusarium* grow on quinoa roots harvested in natural environments near the salt lakes of Chile’s Atacama Desert [60]. González-Teuber et al. [60] showed that inoculation with the endophyte *Penicillium minioluteum* from the Atacama Desert increased canola root biomass by 40% in drought-stressed plants compared to the non-inoculated control plants. The endophytic fungus appeared to have no effect on photosynthesis, stomatal conductance, or plant development. Furthermore, under drought stress, the combination of *P. minioluteum* and quinoa enhanced root growth. Another endophyte, *Piriformospora indica*, was shown to colonize the roots of the quinoa variety ‘Hualhuas’ [60]. The work of Hussin et al. [61] indicated that the quinoa–fungus association reduces certain drought impacts by enhancing crop water content and nutrient uptake, thereby increasing overall biomass, stomatal conductance, leaf moisture, and net photosynthetic rate. The benefit of other root-associated microbes is discussed further in Section 4.1 on salinity. The commercial application of endophytes at the time of seeding is a promising area to explore in field trials with quinoa.

### 3.3. Gene Expression under Drought

In a study on quinoa, the valley variety ‘Ingapirca’ and the Salares variety ‘Ollague’ were used to perform the first RNA sequencing (RNA-seq) transcriptome analysis under drought stress. According to several physiological markers, including stomatal conductance, photosynthetic rate, and stem water potential, ‘Ollague’ was more drought-tolerant than ‘Ingapirca’ [62]. The comparison of RNA-seq data of root samples from these two quinoa varieties under optimal watering conditions and a range of water deficit conditions led to the identification of 462 significantly amplified contigs and 27 candidate genes mainly with unknown functions. Some of these genes are known to have distinct roles, such as *AUR62041909* which functions as a catalyst in the flavonoid biosynthesis pathway, and *AUR62015321*, which is a member of the pathogen-activated protein family associated with lignification [63]. The expression of *AUR62041909* and *AUR62015321* was found to be up-regulated in response to water deficit in the studied quinoa varieties, suggesting that the flavonoid biosynthesis pathway and lignification processes play significant roles in quinoa’s response to drought stress [64]. Since the 1960s, heat-shock proteins (HSPs) have been the subject of extensive scientific investigation in other plant species because of their capacity to respond to a wide range of cellular stressors [65]. In recent years, HSPs have become more well-known as molecular chaperones, preventing the aggregation of related peptides and playing a key role in peptide function [66]. According to their molecular weight, HSPs have been classified into superfamilies such as HSP100, HSP90, HSP70, HSP60, and small heat-shock proteins (sHSPs) [65]. In general, HSP70s are responsible for maintaining crop growth under intense heat (refer to Section 5) and generally perform an important role in crop response to a variety of challenges, such as drought stress [67]. 

Based on the role of HSP70s in Arabidopsis, Liu et al. [68] and Xu et al. [69] identified and characterized sixteen HSP70s (*Cqhsp70s*) in quinoa genomic sequencing. They investigated the expression of thirteen *Cqhsp70s* drought stress genes in response to polyethylene glycol 6000 and showed that their response varied significantly. For instance, at the beginning of drought stress and during the recovery phase, six of the thirteen *Cqhsp70s* genes were down-regulated. In some other cases, the expression of the gene *AUR62024018* remains high throughout the drought treatment. Moreover, a “drop-climb-drop” expression pattern was seen in half of the evaluated genes, resembling the homolog genes in *Arabidopsis*. The transcriptional responses of quinoa during drought stress were examined in another study by Morales et al. [70]. The results showed that the Salares variety ‘R49’ displayed superior drought tolerance compared to the sea-level varieties ‘PRJ’ and ‘BO78’. Pathophysiological markers such as moisture content, electrolyte leakage, and Fv/Fm supported the better performance of ‘R49’. RNA-sequencing yielded 54 million readings under non-drought circumstances, whereas in drought-affected plants, it yielded 51 million readings. All readings were integrated into 150,952 contigs, and 19% of genes were absent in homologous gene libraries. For gene expression analysis, fifteen target genes were selected, some of which were chosen because they were shown to be induced in response to drought stress in other model plants. In particular, the focus was on genes related to the activities of the ABA transport system and ABA production. Target genes that showed variations in read representation in the RNA-seq data were selected. The results showed that plastid-localized CqNCED3a and CqNCDE3b are the only ABA biosynthesis-related genes which were up-regulated in quinoa under drought stress. Additionally, all of the genes that exhibited variations in read representation, including CqLEA (late embryogenesis abundant protein family protein), CqAP2/ERF (integrase-type DNA-binding protein superfamily), CqPP2C (protein phosphatase protein family 2c), CqHSP83 (chaperone protein, protein family HTPG), and CqP5CS (delta 1-pyrroline-5-carboxylate synthase 2), were up-regulated. The CqLEA and CqHSP20 genes showed over 140-fold changes in expression [15]. 

Two previous studies demonstrated that HSPs play a critical function in quinoa’s drought response; hence, quinoa could provide an excellent model plant for studying HSPs under a variety of stress conditions, such as heat, salinity, and drought. Scientific research has become more prominent as a result of efforts to improve plant performance. By comparing quinoa EST databases based on young grain and floral tissues, Maughan’s group [70,71] identified single-nucleotide polymorphisms (SNPs) in quinoa and matched 424 quinoa cDNA-seqs to sequences in available databases. Approximately 67% of the quinoa proteins exhibited substantial homology to Arabidopsis proteins with putative functions, 18% showed no significant matches, 9% had significant homology to Arabidopsis proteins with unknown functions, and 6% shared significant homology with proteins from other plant species. Amplification and sequencing of 34 EST segments on five quinoa germplasm samples derived from a related weedy plant, *C. berlandieri*, indicated a total of 51 SNPs in 20 EST sequences. Further studies by Abd El-Moneim et al. [72] on an additional 113 quinoa accessions detected 14,178 putative SNPs. Two main subgroups that linked with the Andean and Coastal quinoa ecotypes were re-examined in this study. The identification of significant SNPs in quinoa has provided a valuable genomic tool that will be highly beneficial for recently established breeding programs. In addition, an integrated 29-linkage group map for quinoa was constructed as a consequence of the linkage mapping activities. This map covers a genetic distance of 1404 centiMorgans (cM) in total, with a 3.1 cm density for each SNP marker [73].

## 4. Salinity

Before exploring salinity responses in quinoa (Section 4.1), we first provide a brief introduction of saline soils and crop damage. From an agricultural point of view, saline soils are distinguished by a high concentration of soluble, neutral salts which inhibit or reduce the growth of the majority of field crops. However, depending on the particular area or soil type, changes may be made to the threshold criteria used to define salty soils. Selecting a threshold value for saline soil identification requires a thorough understanding of the local soil conditions, climate, and agricultural practices. In certain cases, a lower threshold may be more appropriate when taking into account the vulnerability of particular crops or the possibility of extended salinity-induced soil degradation [74]. An electrical conductivity (EC) of the saturated soil extract exceeding 4 dS m^−1^ at 25 °C has been widely accepted as a standard criterion for identifying saline soils. The 4 dS m^−1^ criterion is still extensively used globally; however, some organizations, such as the Soil Science Society of America, have suggested a lower boundary of 2 dS m^−1^ for defining saline soils. These variations in threshold values can influence how soils are classified as saline across diverse regions or soil types [75].

The typical characteristics of saline soils include the presence of specific ions such as sodium, calcium, magnesium, chlorides, and sulfates, along with the absence of soluble carbonates [76]. Saline soils often contain substantial amounts of gypsum (CaSO_4_ 2H_2_O) within their profiles. Gypsum is an important component of these soils as it helps to reduce salinity problems by exchanging Ca for Na ions, which improves soil structure and water infiltration—two essential properties for salinity management [77]. Soil salinity is widely recognized as an important abiotic stress factor that can seriously restrict agricultural crop productivity [78]. Plants undergo various physiological changes when exposed to salt stress that can have a substantial effect on their growth and productivity [79]. In general, extreme amounts of ROS cause severe crop damage due to the oxidative destruction of proteins, lipids, and DNA. However, low amounts of ROS are essential for signaling [80].

### 4.1. Excessive Salinity and Quinoa Tolerance 

**Figure 3:**
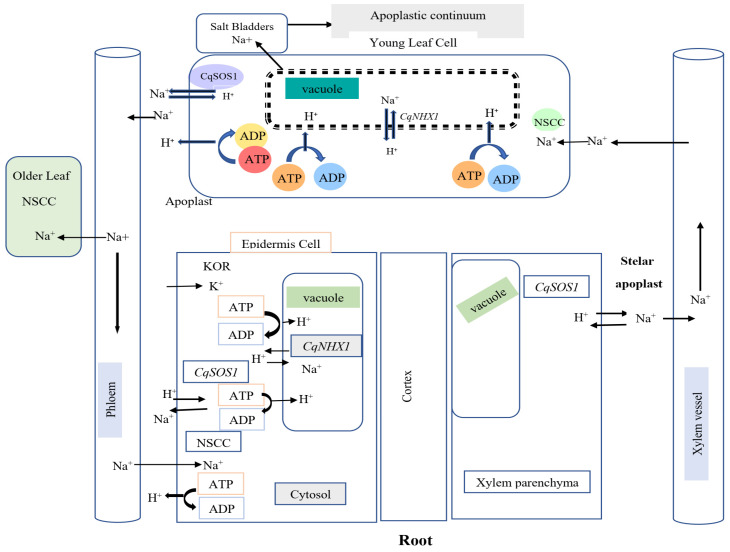
Key pathways for Na uptake, transport, and sequestration in quinoa and the associated gene control mechanisms. Modified from [91]. Abbreviations: sodium (Na), potassium (K), hydrogen (H), adenosine triphosphate (ATP), and adenosine diphosphate (ADP), non-selective cation channels (NSCC), outward rectifying K^+^ channel (KOR).

As a facultative halophyte, quinoa can withstand higher salinities than barley, wheat, corn [81,82], carrots, onions, and asparagus [83]. Quinoa is categorized as an extremophile crop because of its capacity to flourish in conditions marked by low temperatures, drought, and high salinity [84]. Among quinoa genotypes, there is a considerable level of variation in resistance to salt [85]. Traditionally, only genotypes from the Bolivian Salares variety were assumed to exhibit great salt tolerance [7]. The genetic factors responsible for the variation in salt tolerance among different quinoa genotypes include specific genes involved in salt tolerance [16]. These genes, which control processes such as ROS scavenging, protein kinase biosynthesis, and plant hormone signal transduction, are essential for improving salt tolerance in quinoa by facilitating the plant’s adaptation to salinity stress [86]. Moreover, the intricate mechanism of salt tolerance in quinoa is significantly influenced by transcription factors (TFs) and genes involved in ROS scavenging, plant hormone signal transduction, secondary metabolite biosynthesis, and metabolic pathways [87]. However, studies show that salinity tolerance is not necessarily related to the geographic distribution of quinoa. For instance, the salt tolerance levels of varieties from coastal regions of Chile and highland areas outside the Salares ecoregion are comparable or even higher than some other locations [88,89]. In addition, compared to other quinoa cultivars, the wild relative *C. hircinum* exhibited a greater degree of saline tolerance [88]. Typically, quinoa is tolerant of salty soils and can thrive at moderate to high salinity levels. The electrical conductivity of 150 mM NaCl (ca. 15 dS m^−1^) to 750 mM NaCl (ca. 75 dS m^−1^) is the range of salt concentrations that this plant can tolerate [90]. This degree of salinity exceeds the salinity of seawater (>45 dS m^−1^) [91,92]. The Na uptake, transport, and sequestration pathways in quinoa and the associated gene control mechanisms are shown in Figure 3. 

For plants, including halophytes, the most vulnerable period to salinity is the seedling and germination stage [93]. Salt concentrations between 100 and 250 mM NaCl have little effect on the germination rate of most quinoa genotypes [94]. However, concentrations of 150–250 mM NaCl delay the onset of germination [90]. During the germination of quinoa under salt stress, a shift in the metabolism of soluble sugars and invertase activity has been observed [95,96].

One essential process that enables quinoa to tolerate elevated salt concentrations is osmotic adjustment. To maintain cell turgor and promote water uptake in saline environments, this mechanism involves the accumulation of certain osmolytes, such as inorganic ions and organic molecules [16]. Up to 95% of the osmotic adjustment in older leaves and 80–85% in younger quinoa leaves is explained by the buildup of inorganic ions, primarily potassium (K^+^) and chloride (Cl^−^) [97]. High concentrations of these ions accumulate in the vacuole, which helps the plant retain cell turgor and water absorption, which makes it easier for it to withstand salt stress [19]. To further assist with osmotic adjustment, quinoa also accumulates organic osmolytes such as proline, glycine betaine (GBT), and sugars. In particular, GBT can account for as much as 67% of the osmotic adjustment in the cytoplasm [98]. These organic molecules in quinoa may help protect enzymes and cellular structures against the damaging impacts of high salinity [19]. 

The osmotic stress caused by high salt concentration leads to an increase in ABA production in plant roots. Following its transfer to the leaves, ABA functions as a signal to regulate stomatal conductance, thereby affecting photosynthesis by decreasing water loss and reducing CO_2_ uptake [99]. In quinoa, it was found that when the ‘Utusaya’ and ‘Titicaca’ varieties were cultivated at 400 mM NaCl, the CO_2_ assimilation rates decreased by 25% and 67%, respectively, compared to control conditions (distilled water, zero NaCl) [100]. A decrease in the net assimilation rate of photosynthesis was also observed in the ‘Achachino’ variety of quinoa at a Photosynthetic Active Radiation (PAR) level of 1500 μmol m^−2^ s^−1^ [101] under moderate salinity conditions (250 mM NaCl). Also, 500 mM NaCl decreased the net photosynthetic rate by 70% in the ‘Hualhuas’ valley variety [102]. Furthermore, in an experiment with ‘Titicaca’, it was discovered that increasing the water salinity from 100 to 400 mM NaCl decreased the net photosynthesis assimilation rate and seed yield by 48% and 72%, respectively [103]. Moreover, elevated atmospheric CO_2_ (540 ppm) was shown to mitigate the impacts of high salinity by reducing the impact of stomatal restriction on photosynthesis, consequently, decreasing the likelihood of oxidative stress [104]. In addition, the variety ‘Titicaca’, grown in a Mediterranean environment with 22 dS m^−1^ and limited water (three treatments irrigated, with the restitution of 100%, 50%, and 25% of the water necessary to replenish field capacity, with saline water and three treatments irrigated with well water), showed no yield reduction [39,105]. By contrast, under Mediterranean conditions, the ‘Red Head’ variety of quinoa was highly susceptible to salinity (30 dS m^−1^) with impaired photosynthesis [46]. 

Recent studies have explored new strategies to enhance quinoa’s response to salinity stress. For instance, sodium exclusion from the shoot, achieved through restricting Na^+^ transport to leaves, sequestration in leaf vacuoles and bladder cells, and higher Na^+^ accumulation in roots, are critical adaptive strategies that enable quinoa to tolerate high salinity conditions [106].

Halotolerant rhizobacteria and seed priming are being investigated as possible alternatives to improve quinoa’s physiological responses to salinity stress [40,107]. Plant growth-promoting rhizobacteria have been considered for their potential to mitigate the negative effects of salt stress by fixing nitrogen, producing siderophores, dissolving mineral-insoluble phosphate, and generating phytohormones. These approaches present opportunities to strengthen the ability of quinoa to withstand high-salinity environments [108] together with other cutting-edge techniques [109]. Several research endeavors have examined the possibility of halotolerant microorganisms augmenting the salinity tolerance of quinoa. For instance, a study by Yang et al. [40] focused on the interaction between quinoa and halotolerant plant growth-promoting bacteria (*Enterobacter* sp. and *Bacillus* sp.) in saline environments. In addition, studies have demonstrated the ability of halotolerant bacteria isolated from extreme environments to stimulate seed germination and promote quinoa growth under salinity stress [110]. *Bacillus licheniformis* QA1 and *Enterobacter asburiae* QF11 are examples of two other rhizospheric halotolerant phosphate-solubilizing bacteria that can be used to improve plant growth in quinoa under salinity stress [111]. When quinoa was grown in an environment with 300 mM NaCl, both bacterial strains reduced the negative effects of salinity, resulting in a decline in Na^+^ uptake, as well as an improvement in plant–water relations. Furthermore, the same research team illustrated that employing saponin as a seed primer improved germination at 400 mM NaCl, suggesting that saponin priming may be a useful and affordable strategy to enhance quinoa development in high-salinity environments [107]. The findings revealed that the utilization of both hydropriming (treatment with water) and osmo-priming (treatment with polyethylene glycol) significantly improved seed germination rates in ‘Titicaca’ under saline conditions [112]. Moreover, paclobutrazol, an inhibitor of gibberellic acid biosynthesis, has been used to boost quinoa yields while reducing plant height [113]. Under high salinity conditions (400 mM NaCl), foliar-applied paclobutrazol (20 mg L^−1^) enhanced levels of chlorophyll and carotenoids, augmented stomatal density on leaf surfaces, and increased the accumulation of osmo-protectants and antioxidants in both root and leaf tissues of the sea-level variety ‘Pichaman’ [114]. However, there is great concern about the negative environmental effects of paclobutrazol [115]. 

As a facultative halophyte, quinoa may be used to explore processes of salt stress tolerance because of its capacity to withstand high salinity levels [16]. The mechanisms by which quinoa resists salt are as follows: (1) Efficient osmotic modification in leaves by inorganic ion accumulation (Na^+^, K^+^, and Cl^−^) [116,117]; (2) preventing sodium accumulation in the cytosol by effectively controlling Na^+^ sequestration in leaf vacuoles as well as Na^+^ loading in xylem [118]; (3) performing higher tolerance to reactive oxygen species (ROS) [100]; (4) accomplishing better K^+^ retention; and (5) reducing stomatal density, resulting in improved water use efficiency [102]. The investigation of morphological characteristics in quinoa—specifically, stomatal density and the occurrence of epidermal bladder cells (EBCs)—has been a focal point in research exploring responses to salinity stress [90,101,118,119]. The epidermal bladder cells (EBCs) in quinoa are thought to contribute to the plant’s resilience against ultraviolet (UV) radiation by storing betacyanins and flavonoids, compounds known for their UV protective and water regulation properties [120,121]. These specialized cells are believed to play a role in mediating salinity tolerance by storing K^+^ and Na^+^ ions, reducing water loss, and preventing UV damage [122]. A number of studies have highlighted that EBCs are a significant environmental stress response mechanism, especially in areas with high light intensity, as they have been linked to protecting leaves against UV-B radiation [123]. Moreover, it has been demonstrated that EBCs serve functions other than salt accumulation, such as herbivore defense tools [124]. In *Chenopodium* species, similar to quinoa, EBCs serve both structural and chemical roles in plant defense systems, serving as a defensive barrier against herbivorous pests [125]. Quinoa’s EBCs—mainly localized in the leaves, stems, and inflorescences [119]—are principally responsible for storing metabolites, including gamma-aminobutyric acid (GABA), improving potassium (K^+^) retention, and sequestering salt. It has been shown that EBCs accumulate potassium (K^+^) as the major cation in saline soil, far exceeding sodium (Na^+^) quantities [126]. The abundance of EBCs in quinoa remains unchanged in reaction to elevated salinity levels, as indicated by Becker et al. [101] and Orsini et al. [90]. Nonetheless, young leaves have a higher quantity of EBCs in comparison to older leaves [100,127]. Moreover, Bonales-Alatorre et al. [127] reported significant Na^+^ accumulation within the EBCs of young quinoa leaves under saline conditions (400 mM NaCl). The recent completion of a draft quinoa genome for the ‘Real’ Salares variety has facilitated comparative transcriptome analysis of EBCs under salt-treated (100 mM NaCl) and untreated conditions [128]. 

Becker et al. [101], Orsini et al. [90], and Shabala Lana et al. [129] have all addressed the topic of stomatal area and density in quinoa under varied salinity conditions. In ‘Titicaca’, a salinity level of 400 mM NaCl reduced the stomatal density per leaf area across young, intermediate, and old leaves [129]. For the Chilean sea-level variety ‘BO78’, a 750 mM NaCl treatment resulted in a 54% reduction in stomatal density, surpassing the impact observed in the non-treated control group [90]. The study by Shabala et al. [118] using 14 quinoa varieties subjected to 400 mM NaCl revealed a decrease in stomatal densities across all varieties. Opposite results were observed in the ‘Achachino’ variety, which showed that growing the quinoa plants in 250 mM NaCl increased stomatal density by around 18%; nevertheless, the salinity impact decreased the stomatal size [101]. This suggests that, in saline conditions, stomatal density and size might be crucial factors in optimizing water usage efficiency.

### 4.2. Salt Tolerance Mechanisms

The molecular mechanisms underlying salt accumulation by EBCs in quinoa have recently been described [128,130]. In a study, a favorable association was reported between EBC density and salt stress resistance [131]. It has been shown that quinoa crops lacking EBCs are less resistant to salinity [119]. These findings indicate that EBCs play a role in reducing the negative effects of extreme salt stress by accumulating the excess salt in the vacuoles. In addition to salts, EBCs contain plant pigments like betalain and other metabolites [119]. Quinoa EBCs play a part in UV-B stress resistance in addition to salt tolerance [46]. These results suggest that EBCs engage in activities other than salt accumulation. Physiological research has revealed several aspects of EBC function [130], but no research has evaluated the ontogeny of EBCs. However, some putative genes for EBC development have been identified in *Mesophyllum crystallinum* [132,133]. 

In quinoa, increased levels of compatible solutes such as proline and antioxidants are associated with increased salt tolerance potential. However, studies suggest that proline may not have a significant impact on either osmotic adjustment or the tissue tolerance mechanism [134]. Choline (Cho^+^) is a metabolic precursor for glycine betaine and plays a crucial role in the osmotic regulation of salt stress in quinoa. This occurs through the blockage of tonoplast slow vacuolar channels in leaf and root tissues, facilitating the effective sequestration of Na^+^ ions [135]. Polyamines in four Chilean seed cultivars of quinoa were tested in the control group (0 mM NaCl) and 300 mM NaCl conditions. The evaluation of four Chilean cultivars for their seed polyamines under high salinity conditions (300 mM NaCl) showed that the polyamine content decreased significantly, although the ratio of spermidine + spermine/putrescine increased by up to tenfold [136]. Rutin is a non-enzymatic antioxidant that increases quinoa’s tolerance to salt by scavenging hydroxyl radicals [137]. Furthermore, the efficiency of antioxidant enzymes in quinoa is usually influenced by the intensity of salt stress. For instance, it has been found that the type and amount of salts influence the expression of antioxidant enzymes in seedlings of the ‘Titicaca’ quinoa variety [138]. As an illustration, the activity of several antioxidant enzymes including POX, APX, and CAT was significantly boosted by saltwater from the Tyrrhenian Sea [138]. H^+^-ATPase is one of the active transport mechanisms, along with ion channels and co-transporters, that help maintain the proper balance of K^+^ and Na^+^ inside cells. Studies showed that sodium is quickly removed from the cytoplasm [139,140]. In a study on quinoa and *Pisum sativum*, when plants were grown under moderate salinity conditions, the high concentrations of K^+^ in roots and shoots allowed for increased activity of the ion pumps responsible for maintaining the ion balance [141]. Furthermore, the effect of salt stress on the regulation of mitogen-activated protein kinase (MAPK) was investigated in quinoa seedlings and seeds in response to salt stress. Throughout the germination stage, MAPK activity in grains steadily declined from a reasonably high level. MAPK activity usually changes quickly following absorption, regardless of irrigation with pure or salty water. Moreover, in salinized conditions, MAPK activity decreased more rapidly than in non-stressed conditions [142]. 

### 4.3. Salinity and Seed Quality

In field trials conducted in Italy, the quinoa cultivars ‘Titicaca’ and ‘Q52’ were assessed under salt stress conditions (22 dS m^−1^), using different irrigation schedules. Both seed fiber and saponin concentrations decreased in response to the maximum amount of salt, whereas the polypeptide composition remained unchanged [143]. In a study conducted in a lower saline-alkaline environment (6.5 dS/m) in Larissa, Greece, seed protein content was enhanced in eight quinoa varieties [144]. Furthermore, when grown under conditions of 32 dS/m sodium sulfate (Na_2_SO_4_), four sea-level quinoa varieties [CO407D (PI 596293), UDEC-1 (PI 634923), Baer (PI 634918), and QQ065 (PI 614880)] exhibited increased levels of seed protein content [145]. In a separate study conducted in Larissa, Greece, Ca, Mg, Zn, and Mn contents of seed were reduced in quinoa grown in saline-sodic soil [144]. Similar results were reported for the valley variety of quinoa ‘Hualhuas’ in the northwest of Sinai, Egypt, at a salinity level of 17 dS m^−1^. X-ray microanalysis identified significant Na accumulation in the pericarp and embryo tissues of quinoa seeds, while lower levels were detected in the perisperm. Furthermore, the study found that higher salinity levels were linked to an increase in the concentration of essential minerals including Fe [146]. 

Three quinoa varieties (Salares variation ‘R49’, and two sea-level variations ‘VI-1’ and ‘Villarrica’) grown at two salinity levels (100 and 300 mM NaCl) were evaluated for their proteomics and amino acid patterns, total phenols, and antioxidant properties [85]. The results indicated that while all amino acids produced by protein hydrolysis were reduced in the ‘VI-1’ and ‘Villarrica’ varieties, several amino acids in the ‘R49’ variety either increased or remained unchanged as salinity rose. A significant boost in phenolic content was noticed in all three cultivars as the salinity level increased, albeit with a greater portion in ‘R49’. Similarly, ‘R49’ showed a higher increase in overall flavonoids and antioxidant activity [85]. 

### 4.4. Transcriptional Changes under Saline Conditions 

Important transcription factors involved in quinoa’s response to salt were identified by Aloisi et al. [85]. Many other alleles associated with saline stress response in quinoa have also been discovered following the publication of one complete and two approved quinoa genomes [17,147]. Table 1 lists the proven genes and potential genes examined in quinoa under saline conditions. Two genes are principally responsible for the removal of Na^+^ from the cytoplasm. Salt Overly Sensitive 1 (*SOS1*) is one of the important genes encoding a Na^+^/H^+^ antiporter that is present in the cell membrane of the root epidermis and functions to expel Na^+^ out of the cells [148]. Tonoplast-specific Na^+^/H^+^ exchanger 1 (*NHX1*) is another gene that sequesters Na^+^ within the vacuole [149]. The two homoeologous SOS1 loci (cqSOS1A and cqSOS1B) were cloned, sequenced, and described using the Salares variety ‘Ollague’ under saline conditions (300 mM NaCl) [72]. These genes were reportedly activated in the leaf but not in the root cells. Similar results were previously reported for other quinoa varieties grown in 300 mM and 450 mM NaCl solutions [136,150]. 

A salt-tolerant quinoa variety from Chile grown in 300 mM NaCl showed an increased expression of the *CqNHX1* gene in shoots and roots [16]. Furthermore, studies on two Salares varieties (Ollague and Chipaya), as well as one valley variety (CICA) treated with 450 mM NaCl revealed elevated transcription levels of tonoplast intrinsic protein 2 (TIP2) and betaine aldehyde dehydrogenase (BADH) [150]. Notably, the root tissues of Salares-type genotypes had higher levels of BADH, demonstrating the key function of betaine in reducing salt stress in the roots. Furthermore, the results implied the involvement of additional genes in the processes of salt stress response [150].

Ruiz et al. [89] investigated the genes involved in ABA, proline, and polyamine (PA) biosynthesis in two quinoa varieties (R49 and Villarica) grown under 300 mM NaCl. The ABA-related responses served as the basis for the salt adaptation mechanism. For instance, the most significantly stimulated gene was 9-cis-epoxy carotenoid dioxygenase (NCED), which encodes for the primary enzyme involved in the ABA synthesis pathway. In addition, phylogenetic analysis revealed that the quinoa genome contains more gene families implicated in ABA signaling than other Amaranthaceae species [147]. Ortholog genes in ABA production, transport, and sensing in quinoa were identified in saline circumstances [153]. Therefore, it can be concluded that the ABA biosynthesis pathway in quinoa includes genes for neoxanthin synthase (NSY), ABA4, short-chain dehydrogenases/reductases (SDRs), and 11 NCEDs. Quinoa has roughly twice as many of these genes as other diploid crops. It possesses higher numbers of ABA receptors and transportation genes, with 22 ABA receptor pyrabactin-resistant (PYL) family genes and 81 ABC transport group (ABCGs) genes, compared to ten PYL and 34 ABCGs in the ornamental *Amaranthus hypochondriacus* [128]. 

Transcriptome analysis of bladder cells in quinoa compared salt treatment (100 mM NaCl) to untreated circumstances [128]. The results showed that bladder cells expressed more genes involved in energy import and ABA synthesis than the leaf lamina. Anion transporter genes, such as cell anion channels (*SLAH*), nitrate transporter (*NRT*), and chloride channel protein (C1C), as well as cation transporter genes, including NHX1 and K^+^ transporter (*HKT1*), are overexpressed in bladder cells. After the salt treatment, 180 and 525 differently expressed genes were found in leaf lamina and bladder cells, respectively. However, the two tissues shared only 25 genes, suggesting that leaf and bladder cells react differently to salt. Furthermore, genes involved in suberin and cutin synthesis were strongly expressed in bladder cells with increasing salt stress [128]. On the other hand, under saline conditions, genes encoding chloroplast and photosynthetic proteins were significantly down-regulated. The transcript levels of two NCED genes and some short-chain SDR genes were increased by six-fold and 1000-fold, respectively, in bladder cells compared to other leaf cells. Moreover, ABA transporter and ABA receptor genes were reportedly overexpressed in bladder cells. The results suggested that bladder cells may maintain a high degree of ABA homeostasis. The pathway shares responsive neoxanthin with an increase in NCED genes caused by salt and drought stress [128]. 

Using morphological and genome prediction techniques, RNA-seq studies were carried out on quinoa, resulting in the identification of 1413 genes up-regulated in response to salt stress [88]. After eliminating transcription factor proteins, 219 genes were selected and sequenced in 14 quinoa lines including six sea-level, four altiplano, two valley, and two Salares genotypes, along with five *C. berlandieri* and two *C. hircinum* accessions [17]. Using copy number variation (CNV) and the presence of SNPs within the five most salt-tolerant and five most salt-sensitive accessions, 14 candidate genes were identified, with six SNPs found in the first exon of the *AUR62043583* gene (Table 1). As a result, the study revealed 15 additional candidate genes that may result in variations in salt tolerance among quinoa types [88]. 

The Caryopyllales, including the Amarantaceae and quinoa, also contain betalains which are reddish-violet and yellow pigments constructed from tyrosine. These compounds have a role in salt-shock resistance due to their antioxidant properties [151]. Mutagenesis using ethyl methanesulfonate (EMS) on the quinoa variety ‘CQ127’ led to the identification of a gene *CqCYP76AD1-1* involved in the green hypocotyl mutant. This gene was later identified and proven as light-dependent in quinoa hypocotyls. These findings revealed that CqCYP76AD1-1 has a role in betalain production during the quinoa hypocotyl coloring process [154]. Further research on this gene in salt-affected plants can be useful because betalain development might play an important role in quinoa hypocotyl protection. 

Lastly, many genes and mechanisms interact intricately in the complicated process of controlling suberin production and its connection to salt stress tolerance in quinoa [155]. Suberin homeostasis plays a critical function in improving the plant’s resistance to salt stress and is closely linked to the metabolism of fatty acids and carbohydrates [15]. β-Ketoacyl-CoA synthase (*KCS*) is an essential enzyme in the biosynthesis of suberin, acting as a major rate-limiting component through its ability to catalyze the condensation of acyl-CoA with malonyl-CoA [156]. Research has demonstrated that introducing *KCS* genes from different species, such as grape and quinoa, into Arabidopsis can enhance salt stress tolerance. For instance, the expression of *KCS* from quinoa in Arabidopsis stimulated the buildup of very-long-chain fatty acids (VLCFAs) with chain lengths of C22-24, resulting in the accumulation of suberin monomers and enhanced salt tolerance [157]. 

## 5. High-Temperature Effects on Quinoa

Heat stress (HS) is a common abiotic stress that plants experience throughout their growth and development [158]. Widespread agricultural losses have been attributed to heat, sometimes in combination with drought. Before discussing quinoa, in this paragraph, we provide a brief introduction of the topic for crops in general. Thermal stress is defined as an air temperature rise that is higher than the optimal temperature for development over a prolonged time—long enough to cause damage and inhibit development and growth [159]. In general, heat exposure is known to cause diverse reactions in plants, influenced by the heat period and plant growth stage. It also induces changes in morphology, including inhibited shoot and root development, enhanced stem branching, structural modifications such as decreased cell size, increased trichome and stomatal densities, and several other phenological alterations [160]. Heat stress results in changes to photosynthesis, respiration, and carbon metabolism activity at the cellular level. It also causes the denaturation of proteins, increased membrane fluidity, cytoskeleton instability, osmolyte accumulation, enzyme deactivation in both mitochondria and chloroplasts, changes in phytohormones such as ABA, salicylic acid, and ethylene, and the formation of secondary metabolites [161]. The increase in ROS leads to oxidative stress, comparable to drought or salinity [162]. Furthermore, heat-shock proteins (HSPs), which were previously discussed in Section 2, are essential for the heat stress reaction (HSR) in crops, in particular, the HSP70 and HSP90 proteins that are necessary for heat stress tolerance [163]. 

The sensitivity of quinoa to high temperatures has been identified as a significant barrier to its global production [164]. A study conducted by Hinojosa et al. [165] evaluated several physiological parameters including plant growth, seed set, and pollen morphology and viability in two quinoa genotypes QQ74 (PI 614886) and 17GR (Ames 13735) in response to heat stress applied by 40 °C/24 °C day/night temperatures compared to the 22 °C/16 °C control conditions. The results showed that heat stress increases the thickness of the intine and exine layers of the pollen wall and also decreases pollen viability by 30–70%. However, the high-temperature treatment had no effect on seed yield and size or leaf greenness. High temperature, on the other hand, increased the rate of photosynthesis. It can be concluded that quinoa has high plasticity in response to high temperatures, even though high temperatures during anthesis impair pollen viability and pollen wall structure [165]. 

There is a wide range of cardinal temperatures for seed germination in quinoa. Two studies on cardinal temperatures, where the effects of 8–50 °C on ten quinoa cultivars [166] as well as 1–54 °C on another four quinoa cultivars [167] were tested, the optimal germination temperature ranged for the ‘Titicaca’ cultivar and the salaries variant ‘Santa Maria’ between 22 and 35 °C, and for the Salares variant ‘Sajama’ between 18 and 36 °C [167]. Also, in several other studies in Chile [164], Italy [168], Morocco [169], Portugal [170], India [171], and the United States [172], high temperatures reduced the seed yield in quinoa. When maximum air temperatures reached 28 °C during long days, quinoa seed diameter decreased by up to 73% [173]. Furthermore, when high temperatures occurred during flowering, night temperatures between 20 and 22 °C reduced grain yield by 23–31% [164]. Therefore, the flowering and seed fill stages are sensitive to high temperatures, which is a concern given the extent of global warming. Further research is required to identify heat-tolerant genotypes and gene markers for future quinoa breeding programs. Overall, it should be noted that optimizing quinoa germination conditions can enhance the accumulation of bioactive compounds and antioxidant activity in the seeds. Studies have shown that germination at specific temperatures and durations can significantly impact the phenolic content and antioxidant activity of sprouted quinoa seeds [174]. The most significant increase in these compounds occurs between the third and fifth days of germination. The germination process induces the synthesis or consumption of phenolic compounds, leading to an overall elevation in their content. This increase in phenolic compounds is attributed to various metabolic and enzymatic events that occur during germination, which helps protect the seeds against free radicals generated during the process [175]. Additionally, a protocol for in vitro quinoa pollen germination has been developed to understand the impact of various stresses on quinoa fertility and seed yield, highlighting the importance of optimizing germination conditions for assessing pollen viability and fertility in quinoa [176]. Furthermore, research on the cardinal temperatures for seed germination of different quinoa cultivars emphasizes the significance of temperature in determining germination rates and percentages, indicating the need for temperature optimization to maximize germination efficiency [177].

## 6. Other Stressors

Abiotic challenges that have been studied to a lesser extent in quinoa include frost, waterlogging, and exposure to heavy metals. When Jacobsen et al. [178] investigated the response of several quinoa types to cold stress, they found that while plants from the Andean lowlands tended to be more sensitive to frost, cultivars in Peru’s Altiplano tolerated −8 °C for 4 h at the two-leaf stage. For example, under a −8 °C cold stress condition, altiplano varieties ‘Witulla’ and ‘Ayara’ had a crop fatality rate of 4.17% after 4 h, while ‘Quillahuaman’ valley variants showed 25% and 50% mortality after 4 and 6 h, respectively. In addition, quinoa was even more susceptible to cold stress during flowering: an exposure to −4 °C for 4 h decreased grain yield by 56% in ‘Quillahuaman’ and 26% in ‘Witulla’ [178]. Further studies on ‘Witulla’ and ‘Quillahuaman’ revealed that proline and high sugar concentrations, particularly in “Witulla,” are crucial in preventing freezing damage [15,178]. Therefore, proline and soluble sugar contents, such as sucrose, might be utilized to predict frost resistance. 

Crops cannot develop without water; however, too much water can have a negative influence on plant growth. Particularly, prolonged waterlogging prevents photosynthesis and stimulates the creation of toxic chemicals that cause plant mortality [179]. Global climate change has led to a rise in the frequency and severity of rainstorms and floods. Plants suffer from total or partial flooding stress in flood-prone/waterlogged circumstances, which reduces crop yields [180]. In general, crop growth and development are greatly hindered by excessive water [181,182]. Therefore, developing flood/waterlog-resistant crops such as quinoa is an effective strategy to help combat climate change and ensure food security [183]. In a study using the altiplano variety ‘Sajama’ under controlled conditions, waterlogging showed multiple adverse consequences including (1) reduced plant biomass; (2) decreased chlorophyll a and chlorophyll b contents; and (3) increased concentrations of soluble carbohydrates [184]. Under field conditions in Brazil, the ‘BRS Piabiru’ quinoa variety produced the highest leaf measurement (maximum leaf retention, maximum leaf area) values when plants were cultivated in a 563 mm water irrigation regime. However, 647 mm irrigation resulted in a decline in leaf function, demonstrating that quinoa is sensitive to excessive moisture [185]. 

The concentration of heavy metals that accumulate in the shoot of quinoa varies among genotypes. For instance, PI 587173, PI 478410, Ames 22158, and *C. giganteum* CHEN 86/85 varieties of quinoa preferentially accumulated higher levels of Zn, Cr, Ni, and Cd, in leaves, whereas Ni, Cr, and Zn were absorbed more efficiently by quinoa PI 510536 and Ames 22156. Furthermore, the altiplano type of quinoa, ‘Quinoa de Quiaca—PI 510532’ hyperaccumulated Cd, Cu, and Pb according to research conducted in a polluted urban ‘brownfield’ in Vancouver, Canada [186]. The accumulation of heavy metals in seeds of quinoa plants cultivated in contaminated or serpentine-rock-derived soils may cause a risk to human health [186]. Leaves of the sea-level variety “Regalona” are able to withstand up to 1 mM external chromium (III) chloride (CrCl_3_), and this activated tocopherol production and increased tyrosine aminotransferase levels in the plants [187]. At 5 mM Cr(III), there was considerable oxidative damage, resulting in high levels of proline and hydrogen peroxide [187]. Identifying useful molecular markers for heavy metal tolerance in chenopods could facilitate alternative, non-food applications for quinoa, such as phytoremediation of contaminated soils. 

## 7. Conclusions and Prospects

Quinoa has been utilized as a model plant for research on salinity tolerance across 86 halophyte species [85]. There has been much greater emphasis on quinoa’s response to drought and salinity than on extreme temperatures or exposure to heavy metals. Quinoa has a high level of resistance to abiotic stresses, as evidenced by the physiological, chemical, and structural adaptations of several quinoa varieties to different abiotic stressors in field and laboratory conditions. It appears that sensitivity and adaptability are genetically regulated, and considerable breakthroughs in breeding efforts have been made as a result of the entire genome sequencing of quinoa, including the identification of transcription factors controlling anti-nutritional triterpenoid saponins, genetic improvement of agronomic traits, genetic diversity, the development of genomics-enabled breeding tools, the identification of genetic markers associated with agronomically important traits, and the application of novel molecular methods. Among the most important characteristics of quinoa, its hypersaline resistance distinguishes it from other crops such as wheat, rice, barley, and maize. The loss of productive arable land is accelerating due to salinization, excessive heat, and severe drought. Producers have started to search for halophytic- and abiotic-tolerant crops, like quinoa, which can thrive in these conditions. Quinoa has emerged as an essential model crop because of its newly sequenced genomes and remarkable resilience to salt stress. As new genes are discovered in quinoa, the confirmation of gene function is required, and this will support advanced breeding programs in the future. Moreover, because quinoa typically exhibits a strong, genetically-dependent response to salinity, breeders may employ a range of quinoa genotypes to produce distinctive saline-tolerant cultivars with high seed yield and other beneficial traits. Another distinguishing property of quinoa seed is its high nutritional content, including vital amino acids and minerals that remain stable under abiotic stress conditions. Quinoa is a very adaptable plant since its seeds and leaves can be used as food, and the aboveground biomass can be utilized as livestock feed or as covering vegetation. Quinoa has been identified as a candidate for phytoremediation due to its ability to accumulate and tolerate heavy metals. Preliminary studies have shown that quinoa can remove heavy metals from contaminated soil. Whether quinoa is a viable option for phytoremediation will depend on the outcomes of further field studies. 

After many decades of applied research, there are still many unanswered questions concerning the interaction of quinoa with abiotic stresses. Future scientific investigations should concentrate on the genetic foundations and processes that underline how quinoa’s tolerance to abiotic stress affects its chemical makeup. This additional knowledge will enable quinoa breeders to better screen the quinoa germplasm and breed new varieties that are adaptable to a wide range of ecological circumstances, facilitating increased global plantings of quinoa. Furthermore, recent research on the connection between quinoa and related species may yield novel genetic combinations with promising breeding prospects in harsh environments. 

## Figures and Tables

**Table 1:** Genes identified as potential candidates contributing to salinity tolerance in quinoa.

Genes	Annotations and Varieties Evaluated	Concentration of Salt	Reference
Salt Hypersensitive 1 (*CqSOS1a*, *CqSOS1b*)	Sal variety ‘Ollague’, up-regulated in leaves but not in roots	300 mM NaCl	[150]
	‘PRJ’, ‘PRP’, ‘UDEC9’, and ‘B078’ are sea-level varieties. More strongly up-regulated in shoots more than in roots	450 mM NaCl	[96]
	Valley variety ‘Cica’ and Salares ‘Ollague’ and ‘Chipaya’ varieties; up-regulated in leaves	450 mM NaCl	[151]
Na^+^/H^+^ exchanger 1 (*CqNHX1*)	Sea-level varieties ‘PRJ’, ‘PRP’, ‘UDEC9’, and ‘B078’; were up-regulated in roots and shoots	450 mM NaCl	[96]
	Valley variety ‘Cica’, as well as the Salares ‘Ollague’ and ‘Chipaya’; leaf and shoot up-regulation	300 mM NaCl	[151]
Betaine aldehyde dehydrogenase (*BADH*)	Valley variety ‘Cica’ and the Salares varieties ‘Ollague’ and ‘Chipaya’; leaf up-regulation	450 mM NaCl	[151]
ABA-related: 9-cis-epoxycarotenoid dioxygenase (*NCED*) ABA-binding factors (*ABF3*) Pyrabactin resistant (*PYR*, *PYL*) β-glucosidase homologues (*BG1*) Polyamine-related Arginine decarboxylase (*ADC1*, *ADC2*) Spermidine synthase (*SPDS1*) S-adenosylmethionine decarboxylase (*SAMDC*) Spermine synthase (*SPMS*) Diamine oxidase (*DAO*) Ion homeostasis-related *CqSOS1a* *CqNHX* K^+^ transporter (*HKT*) Growth: Cyclin D3 (CycD3) Β-Expansion (βEXP1) Stress-related genes Responsive to desiccation 22 (*RD22*) Pyrroline-5-carboxylate (*P5CS*) Transcription factors Dehydration- responsive-element binding protein 2A (*DREB2a*)	Salar variety ‘R49’ and sea-level variety ‘Villarica’	0–120 h with 300 mM NaCl	[89]
	Salar variety ‘R49’ has an early up-regulation of ion homeostasis genes and polyamine-related genes		
	The sea-level variety ‘Villarica’ expresses highly on NCED, RD22, and DREB2a		
Pyrabactin-resistant (PYR, PYL) Serine/threonine kinases (SnRK2)	‘Kd’ is an inbreeding quinoa accession. Quinoa has two phylogenetically related PYR genes as well as two SnRK2 genes	300 mM NaCl	[152]
Copy number of main genes in salinity conditions: 9-cis-epoxycarotenoid dioxygenase (NCED), 11 genes Neoxanthin synthase (NSY), 7 genes ABA4, 2 genes Short-chain dehydrogenases/reductases (SDRs), 37 genes Pyrabactin- resistant (PYL) family, 22 genes Na^+^/H^+^ exchanger, 11 genes ABC transports group (ABCGs), 81 genes Zeaxanthin epoxidase (ZEP), 2 genes Violaxanthin de-epoxidase (VDE), 2 genes Cell anion channels (SLAH), 6 genes Nitrate transporter (NRT), 12 genes Chloride channel protein (C1C), 10 genes Hemoglobin family (HB), 8 genes H^+^ ATPase (AHA), 20 genes Glucose transporter (GLUT), 68 genes	Salar variety ‘Quinoa Real’ (genome and mRNA sequencing)	100 mM NaCl	[129]
	5020 Quinoa Variety (mRNA sequencing in EBCs)		
	EBC is a photosynthetically nonactive tissue that is extremely active in ion transport, cell wall synthesis, and wax synthesis		
Transmembrane domains genes: WAKL8 Wall-associated receptor kinase-like 8 (AUR62006689) (AUR62029668) At1g21890 WAT1-related protein At1g21890 (AUR62039756) At1g67300 Probable plastidic glucose transporter 2 (AUR62021463) CYP75B1 Flavonoid 3′- monooxygenase (AUR62007451) psbD Photosystem II D2 protein (AUR62039871) CER1: Protein ECERIFERUM (AUR62043781) (AUR62043583) AAP6 Amino acid permease 6 (AUR62034957) SULTR1;1 Sulfate transporter 1.1 (AUR62011984) SULTR3;4 Probable sulfate transporter 3.4 (AUR62021522) SULTR3;4 Probable sulfate transporter 3.4 (AUR62016440) CNGC7 Putative cyclic nucleotide-gated ion channel 7 (AUR62004478) DTX14 Protein DETOXIFICATION 14 (AUR62002768) SULTR3;4 is a possible sulfate transporter. 3.4 TMK1 Receptor protein kinase 1 (AUR62041961)	14 quinoas (6 sea-level, 4 altiplano, 2 valley, and 2 Salares varieties) 5 *C. berlandieri* and 2 *C. hircinum* accessions	300 mM NaCl	[88]

## Data Availability

Not applicable.
